# Autoantibodies in Serum of Systemic Scleroderma Patients: Peptide-Based Epitope Mapping Indicates Increased Binding to Cytoplasmic Domains of CXCR3

**DOI:** 10.3389/fimmu.2018.00428

**Published:** 2018-03-22

**Authors:** Andreas Recke, Ann-Katrin Regensburger, Florian Weigold, Antje Müller, Harald Heidecke, Gabriele Marschner, Christoph M. Hammers, Ralf J. Ludwig, Gabriela Riemekasten

**Affiliations:** ^1^Department of Dermatology, University of Lübeck, Lübeck, Germany; ^2^Lübeck Institute of Dermatological Research, University of Lübeck, Lübeck, Germany; ^3^Department of Rheumatology and Clinical Immunology, Charité University Hospital, Berlin, Germany; ^4^Department of Rheumatology, University of Lübeck, Lübeck, Germany; ^5^CellTrend GmbH, Luckenwalde, Germany; ^6^Priority Area Asthma & Allergy, Research Center Borstel, Airway Research Center North (ARCN), German Center for Lung Research (DZL), Borstel, Germany

**Keywords:** autoantibodies, CXCR3, peptide array, systemic sclerosis, G protein-coupled receptor

## Abstract

Systemic sclerosis (SSc) is a severe chronic autoimmune disease with high morbidity and mortality. Sera of patients with SSc contain a large variety of autoantibody (aab) reactivities. Among these are functionally active aab that bind to G protein-coupled receptors (GPCR) such as C-X-C motif chemokine receptor 3 (CXCR3) and 4 (CXCR4). Aab binding to the N-terminal portion of these two GPCRs have been shown to be associated with slower disease progression in SSc, especially deterioration of lung function. Aabs binding to GPCRs exhibit functional activities by stimulating or inhibiting GPCR signaling. The specific functional activity of aabs crucially depends on the epitopes they bind to. To identify the location of important epitopes on CXCR3 recognized by aabs from SSc patients, we applied an array of 36 overlapping 18-20mer peptides covering the entire CXCR3 sequence, comparing epitope specificity of SSc patient sera (*N* = 32, with positive reactivity with CXCR3) to healthy controls (*N* = 30). Binding of SSc patient and control sera to these peptides was determined by ELISA. Using a Bayesian model approach, we found increased binding of SSc patient sera to peptides corresponding to intracellular epitopes within CXCR3, while the binding signal to extracellular portions of CXCR3 was found to be reduced. Experimentally determined epitopes showed a good correspondence to those predicted by the *ABCpred* tool. To verify these results and to translate them into a novel diagnostic ELISA, we combined the peptides that represent SSc-associated epitopes into a single ELISA and evaluated its potential to discriminate SSc patients (*N* = 31) from normal healthy controls (*N* = 47). This ELISA had a sensitivity of 0.61 and a specificity of 0.85. Our data reveals that SSc sera preferentially bind intracellular epitopes of CXCR3, while an extracellular epitope in the N-terminal domain that appears to be target of aabs in healthy individuals is not bound by SSc sera. Based upon our results, we could devise a novel ELISA concept that may be helpful for monitoring of SSc patients.

## Introduction

Systemic sclerosis (SSc) is a severe chronic autoimmune disease with increased mortality, mainly due to affections of the lungs (1, 2). It is characterized by aa pathogenic triad of small vessel vasculopathy, dysregulation of the innate and adaptive immune system and generalized fibrosis of multiple organs (1, 3).

Sera of patients with SSc contain a large variety of autoantibodies (aab) such as anti-nuclear ab (ANA) directed against Scl-70, RNA polymerase 3, and centromere proteins (4). Recently, the presence of functionally active aab that bind to G protein-coupled receptors (GPCRs) such as angiotensin 1 receptor and endothelin A receptor, C-X-C motif chemokine receptor 3 (CXCR3), and 4 (CXCR4) has gained increasing interest (5–9). Interestingly, however, aab directed against GPCRs are found in both healthy individuals and SSc patients (9). Further, they appear to be important in glaucoma, cardiac diseases, preeclampsia, Alzheimer’s disease, Sjögren’s syndrome, renal diseases, renal transplantation, and some cases of metabolic syndrome (10–12). Interestingly, some of these anti-GPCR antibodies are not necessarily associated with clinical worsening of disease. For example, high concentrations of anti-CXCR3 aabs were found to predict a more benign clinical course of pulmonary disease in SSc (7), which is in contrast to anti-angiotensin and anti-endothelin receptor antibodies (13, 14). Of note, in the above mentioned study, an N-terminal extracellular domain fragment of CXCR3 was used to determine antibody binding (7).

CXCR3 is a GPCR that is expressed by activated naïve T cells, Th1-type CD4^+^ T cells, effector CD8^+^ T cells as well as by innate-type lymphocytes (15). It contains an extracellular N-terminus, seven transmembrane domains, and an intracellular C-terminal domain (16). CXCR3 features a series of rhodopsine-like motifs, which are shared among many GPCRs. The role of CXCR3 receptor in connective tissue diseases is supported by the finding that CXCR3^+^ CD4^+^ T cells are enriched in kidneys and urine of patients with systemic lupus erythematosus (17).

The functional activity of aab like those against CXCR3 is crucially dependent on their respective epitopes (9, 18–20). However, it is currently not known if anti-CXCR3 aabs target specific epitopes when comparing SSc patients to healthy controls. To address this knowledge gap, we applied a peptide array to screen a set of SSc patient sera in comparison to healthy controls.

## Materials and Methods

### Patients and Controls

In this study, sera of patients with SSc (*N* = 32, Table 1) were compared to sera of healthy blood donors (*N* = 65). The age of SSc patients ranged from 38 to 76 years, with 15 female and 5 male subjects. The age of healthy blood donors ranged from 20 to 60 years, with a larger amount of male subjects. The diagnosis in SSc patients was established according to the ACR/EULAR classification criteria for SSc (1). In sera of all SSc patients, positive titers of anti-CXCR3 ab were detected (Table 1). Unselected healthy blood donor sera were obtained from the Institute of Transfusion Medicine at the University of Lübeck. All studies with human materials followed the ethical principles established by the Declaration of Helsinki and were approved by the local ethics committee (AZ16-199). All human participants gave their written informed consent.

**Table 1:** Systemic sclerosis patient overview.

#	ID	SSc variant^1^	ENA^2^	CXCR3 Ab (U/ml)
1	51_SSC	limited	anti-Scl70	15.213
2	80_SSC	limited	anti-Scl70	3.775
3	88_SSC	diffuse with myositis overlap	anti-Ro52	5.156
4	96_SSC	diffuse	anti-Scl70	4.008
5	110_SSC	limited	anti-CENP-B	5.023
6	117_SSC	limited	anti-CENP-B	6.950
7	202_SSC	limited	anti-Scl70	15.399
8	215_SSC	diffuse	anti-Scl70	3.853
9	216_SSC	limited	anti-CENP-B	3.805
10	226_SSC	limited	anti-CENP-B	5.295
11	235_SSC	limited	anti-CENP-B	6.982
12	279_SSC	limited	anti-RNP/sm+	7.984
			anti-Sm+	
			anti-PmScl75	
13	313_SSC	diffuse	anti-Scl70	6.080
14	327_SSC	limited	anti-CENP-B	7.770
15	332_SSC	limited	anti-Scl70	4.350
16	335_SSC	diffuse	anti-Scl70	4.828
17	368_SSC	limited	anti-Scl70	4.299
18	373_SSC	limited	anti-CENP-B	4.084
19	383_SSC	diffuse	dense fine speckled pattern	8.065
20	384_SSC	limited	anti-Scl70	5.381
21	SKL011	limited	anti-CENP-B	6.577
22	SKL024	limited	anti-CENP-B	2.948
23	SKL028	diffuse	anti-Scl70	2.876
24	SKL033	diffuse	n.d.	12.163
25	SKL034	diffuse	anti-Scl70	6.542
26	SKL102	limited	anti-CENP-B	25.575
27	SKL103	UCTD	n.d.	18.678
28	SKL109	limited	anti-CENP-B	4.042
29	SKL111	MCTD	n.d.	13.025
30	SKL117	limited	anti-CENP-B	38.758
31	SKL157	limited	anti-CENP-B	11.802
32	SKL224	limited	anti-CENP-B	3.717

*Limited or *diffuse* systemic sclerosis, undifferentiated connective tissue disease (*UCTD*), mixed connective tissue disease (MCTD)*.

*Reactivity against specific extractable nuclear antigens (ENA). n.d., not differentiated. All SSc patients were ANA-positive*.

### Human Anti-CXCR3 IgG ELISA

Anti-CXCR3 IgG aabs were measured by a commercially available sandwich ELISA kit from CellTrend GmbH (Luckenwalde, Berlin, Germany) (7). The antigen in this assay is a recombinant fragment comprising the N-terminal potion of CXCR3. Measurements were performed according to the manufacturer’s instructions. The kit includes a standard to determine autoantibody concentrations (in U/ml).

### Human CXCR3 Peptides

Thirty-six biotinylated 20mer peptides covering the entire sequence of CXCR3 (isoform 1, aa 1-368, UNIPROT accession ID P49682 (CXCR3_HUMAN), URL: www.uniprot.org, last accessed 9/25/2017) were synthesized by peptides & elephants (Henningsdorf, Germany), with overlaps of 10 aa to the corresponding peptides upstream and downstream the protein sequence (Table 2). Peptides were delivered as lyophilized trifluoro-acetate salts and dissolved according to hydrophobicity and isoelectric point (pI) in NaOH, HCl, or DMF. Dissolved peptides were further diluted in phosphate-buffered saline (PBS), pH 7.2.

**Table 2:** Characteristics of peptide fragments of CXCR3.

#	Sequence^1^	Length	Residues^2^	MW^3^	Hydrophobicity^4^	pI^5^	Charge^6^
1	MVLEVSDHQVLNDAEVAALL	20	1–20	2392.3	0.72	3.74	−3.92
2	LNDAEVAALLENFSSSYDYG	20	11–30	2441.0	−0.13	3.30	−4.09
3	ENFSSSYDYGENESDSCCTS	20	21–40	2486.8	−1.26	3.23	−5.18
4	ENESDSCCTSPPCPQDFSLN	20	31–50	2435.7	−0.96	3.30	−4.23
5	PPCPQDFSLNFDRAFLPALY	20	41–60	2536.5	−0.06	4.11	−1.14
6	FDRAFLPALYSLLFLLGLLG	20	51–70	2464.9	1.50	6.33	−0.09
7	SLLFLLGLLGNGAVAAVLLS	20	61–80	2188.8	2.04	6.10	−0.09
8	NGAVAAVLLSRRTALSSTDT	20	71–90	2228.4	0.31	10.40	0.91
9	RRTALSSTDTFLLHLAVADT	20	81–100	2413.6	0.20	7.55	0.08
10	FLLHLAVADTLLVLTLPLWA	20	91–110	2446.1	1.82	5.29	−0.92
11	LLVLTLPLWAVDAAVQWVFG	20	101–120	2476.0	1.63	3.75	−1.09
12	VDAAVQWVFGSGLCKVAGAL	20	111–130	2215.7	1.16	6.16	−0.14
13	SGLCKVAGALFNINFYAGAL	20	121–140	2255.0	1.06	8.52	0.86
14	FNINFYAGALLLACISFDRY	20	131–150	2538.0	0.90	6.16	−0.14
15	LLACISFDRYLNIVHATQLY	20	141–160	2579.6	0.75	7.35	0.03
16	LNIVHATQLYRRGPPARVTL	20	151–170	2500.9	−0.11	12.20	3.07
17	RRGPPARVTLTCLAVWGLCL	20	161–180	2408.2	0.62	10.52	2.81
18	TCLAVWGLCLLFALPDFIFL	20	171–190	2501.5	2.07	3.75	−1.18
19	LFALPDFIFLSAHHDERLNA	20	181–200	2551.7	0.31	5.36	−1.75
20	SAHHDERLNATHCQYNFPQV	20	191–210	2592.7	−1.13	6.78	−0.64
21	THCQYNFPQVGRTALRVLQL	20	201–220	2571.2	−0.20	9.50	2.03
22	GRTALRVLQLVAGFLLPLLV	20	211–230	2375.3	1.50	12.50	1.91
23	VAGFLLPLLVMAYCYAHILA	20	221–240	2404.6	1.93	7.35	0.02
24	MAYCYAHILAVLLVSRGQRR	20	231–250	2546.0	0.51	10.13	3.02
25	VLLVSRGQRRLRAMRLVVVV	20	241–260	2545.8	0.85	13.10	4.91
26	LRAMRLVVVVVVAFALCWTP	20	251–270	2471.0	1.85	10.53	1.86
27	VVAFALCWTPYHLVVLVDIL	20	261–280	2497.5	1.92	5.29	−0.97
28	YHLVVLVDILMDLGALARNC	20	271–290	2454.4	1.21	5.41	−0.97
29	MDLGALARNCGRESRVDVAK	20	281–300	2388.1	−0.36	8.55	0.86
30	GRESRVDVAKSVTSGLGYMH	20	291–310	2374.9	−0.43	9.30	1.08
31	SVTSGLGYMHCCLNPLLYAF	20	301–320	2415.2	0.85	7.25	−0.02
32	CCLNPLLYAFVGVKFRERMW	20	311–330	2672.8	0.50	8.80	1.81
33	VGVKFRERMWMLLLRLGCPN	20	321–340	2644.2	0.25	11.38	2.86
34	MLLLRLGCPNQRGLQRQPSS	20	331–350	2492.9	−0.49	12.20	2.86
35	QRGLQRQPSSSRRDSSWSET	20	341–360	2574.1	−2.01	12.02	1.91
36	SRRDSSWSETSEASYSGL	18	351–368	2229.0	−1.27	4.43	−1.09

*Sequence of peptides in one letter format. Each peptide listed is biotinylated at the N-terminus*.

*Residues are numbered according to Uniprot entry P49682 (CXCR3_HUMAN) isoform 1*.

*Molecular weight as determined by mass spectrometric quality report of manufacturer*.

*Hydrophobicity scores were calculated with the function *hydrophobicity* of R package *peptides* by the method of Kyte and Doolittle (21)*.

*pI (isoelectric point) values were calculated with the function *pI* of R package *peptides* with the default EMBOSS method*.

*The peptide sum charge was calculated a pH 7.2 with the function charge of R packages peptides, with the method Stryer*.

### Peptide-Based ELISA

Biotinylated peptides were diluted in PBS (pH 7.2) containing 0.05% Tween-20 (PBS-T) to a concentration of 20 µg/ml and incubated for 1 h at room temperature with streptavidine-coated 96-well plates (Immobilizer Streptavidin F96 Clear; Thermo Fisher Scientific p/a Nunc, Langenselbold, Germany). For each well only one peptide was used. As positive control biotinylated anti-human IgG1 (Thermo Fisher Scientific, p/a Invitrogen, Carlsbad, CA, USA) was used, negative control wells were left empty. Each control and peptide was placed twice on a single 96-well plate, to allow for testing of two individual sera. We combined one SSc serum (*N* = 32) with one control serum (*N* = 30 of the 65) to aid comparability. After automatic washing of ELISA plates with an Columbus Pro plate washer (TECAN Group AG, Männedorf, Schweiz), plates were incubated for 1 h at room temperature on an orbital shaker containing 100 μl/well SSc patient or control sera diluted 1:100 in PBS-T. Subsequently, after another automatic washing step plates were incubated for 1 h at room temperature with a peroxidase-conjugated polyclonal anti-human IgG antibody (DAKO, Hamburg, Germany), diluted 1:1000 in PBS-T. 1-step™ Turbo-TMB-ELISA (Thermo Fisher Scientific, p/a Pierce Biotechnology, Rockford, USA) was used as chromogenic substrate with 0.5 M H_2_SO_4_ as stop solution. The optical density at 450 nm (OD450) was recorded by a VICTOR 3™ (PerkinElmer Inc., Waltham, MA, USA) reader.

Complete and annotated ELISA readout data used for this analysis is made available for public access (Supplementary Material).

### Mixed Peptide ELISA

Peptides 17, 24, 25, 33, and 34 were combined in PBS-T with a total concentration of 20 µg/ml and incubated with streptavidine-coated 96-well plates (Immobilizer Streptavidin F96 Clear; Thermo Fisher Scientific p/a Nunc, Langenselbold, Germany), as described above. All SSc patient sera, 16 healthy controls used for peptide mapping and 31 previously not used healthy controls were measured as duplicates. The experimental procedures for this assay were performed with exact timing, to ensure comparability of OD 450 nm values between plates.

### *In Silico* Prediction of Epitopes

We used two different software approaches to predict continuous epitopes in the CXCR3 sequence. *Antigenic* from the *EMBOSS* package available online at http://www.bioinformatics.nl/cgi-bin/emboss/antigenic (last visited 2/9/2018) with a window size of 6 amino acids. *Antigenic* is based on sliding window averaging antigenicity scores of amino acids in the sequence of proteins. The other software, ABCpred, available at http://crdd.osdd.net/raghava/abcpred/ABC_submission.html (last visited 2/10/2018), is based upon a trained neural network that determines a score for subsequences of protein sequences in a sliding window approach. We used a window size of 20 amino acids and a minimum score of 0.8.

### Statistical Analysis of Peptide-Mapping Data

For isolation of specific binding signals from peptide-mapping ELISA data, a mixed effects model was established using R open source statistical software (URL: http://www.r-project.org/, last visited 9/25/2017) together with the framework provided by R package *INLA* (URL: http://www.r-inla.org/, last visited 9/25/2017) (22–24). Peptide properties were calculated with R package *peptides*. 1.1 was determined and the normalized value $\hat{x}=\left(x-u\right)/\left(o-u\right)$ was calculated.

To normalize the ELISA signal *x* per plate, an upper limit o=max(x)×1.1 and a lower limit u=min(x)/1.1. The logit value of ELISA signals was calculated as logit(x)=ln(x^1−x^). For sample runs with a variance below 25% quantile were not further processed.

To analyze the ELISA signals, a mixed effects model was set up using the INLA framework with logit(*x*) as the dependent variable and isoelectric point (pI) and hydrophobicity of peptides as fixed effects with the default vague prior distribution settings. As random effects, plate ID and serum ID were included with an *iid* model, and the peptide numbers with a special autoregressive model of order 1 (*ar1*). To separate the SSc autoantibody-binding signal, a simple *ar1* model was combined with a weighted *ar1* model. For SSc patients, the weighting factor was set to 1, for healthy controls it was set to 0. For all random effect models, the default vague prior distribution of the hyper-parameter was chosen.

A Bayesian analog of a *p* value (*p*_Bayes_) was calculated as described (25, 26). Briefly, for a given posterior distribution of regression coefficients, the largest α ∈ [0;1] was determined such that the α highest-posterior density credibility interval does not contain the point 0. The *p*_Bayes_ was then calculated as *p*_Bayes_ = (1−α).

The R code and data set specifications are described in detail in the Supplementary Methods and Supplementary Information in Data Sheet S1 in Supplementary Material.

### Statistical Analysis of Mixed-Peptide ELISA Data

Mixed peptide data were evaluated as raw OD450 data using R open source statistical software with additional packages *beeswarm* for visual representation of data (stacked scatter plots) and *ROCR* for receiver operator characteristic (ROC) analysis. The cut-off value was calculated by optimization of Matthew’s correlation coefficient (MCC) (27). The MCC was calculated as:

$$\text{MCC=}\frac{\text{TP}\times \text{TN-FP}\times \text{FN}}{\sqrt{\left(\text{TP+FP}\right)\left(\text{TP+FN}\right)\left(\text{TN+FP}\right)\left(\text{TN+FN}\right)}}$$

with TP being the number of true positives, TN the number of true negatives, FP the number of false positives, and FN the number of false negatives.

For performance parameters sensitivity, specificity, positive likelihood ratio (LR)+, and negative LR−, 95% binomial confidence intervals (95% CI) were calculated using the Clopper–Pearson method.

## Results

### Epitope Mapping Localizes Epitopes of Autoantibodies in SSc in Intracellular Regions of CXCR3

Aab recognizing an N-terminal extracellular fragment of CXCR3 ranging from 3.8 until 15.4 U/ml were detected by ELISA in sera of SSc patients (Table 1). A peptide array covering the whole aa sequence of CXCR3 (Table 2) was applied to determine epitopes of CXCR3 targeted by anti-CXCR3 contained in serum of patients with SSc and controls. The measurements were analyzed in comparison to those from sera of healthy control blood donors. Raw ELISA signal data of the peptide array is shown in Figure S1 in Supplementary Material.

Linear peptides, by design, do not have the same conformation as compared to a fully folded protein further stabilized by a cell membrane. This is a potential source of non-specific binding signal variation, which needs to be taken into account appropriately during data analysis. Additional variation is generated because sera of patients and healthy controls do not only contain ab directed against CXCR3, but a whole set of different antibodies that may bind non-specifically to the peptides.

**Figure 1:**
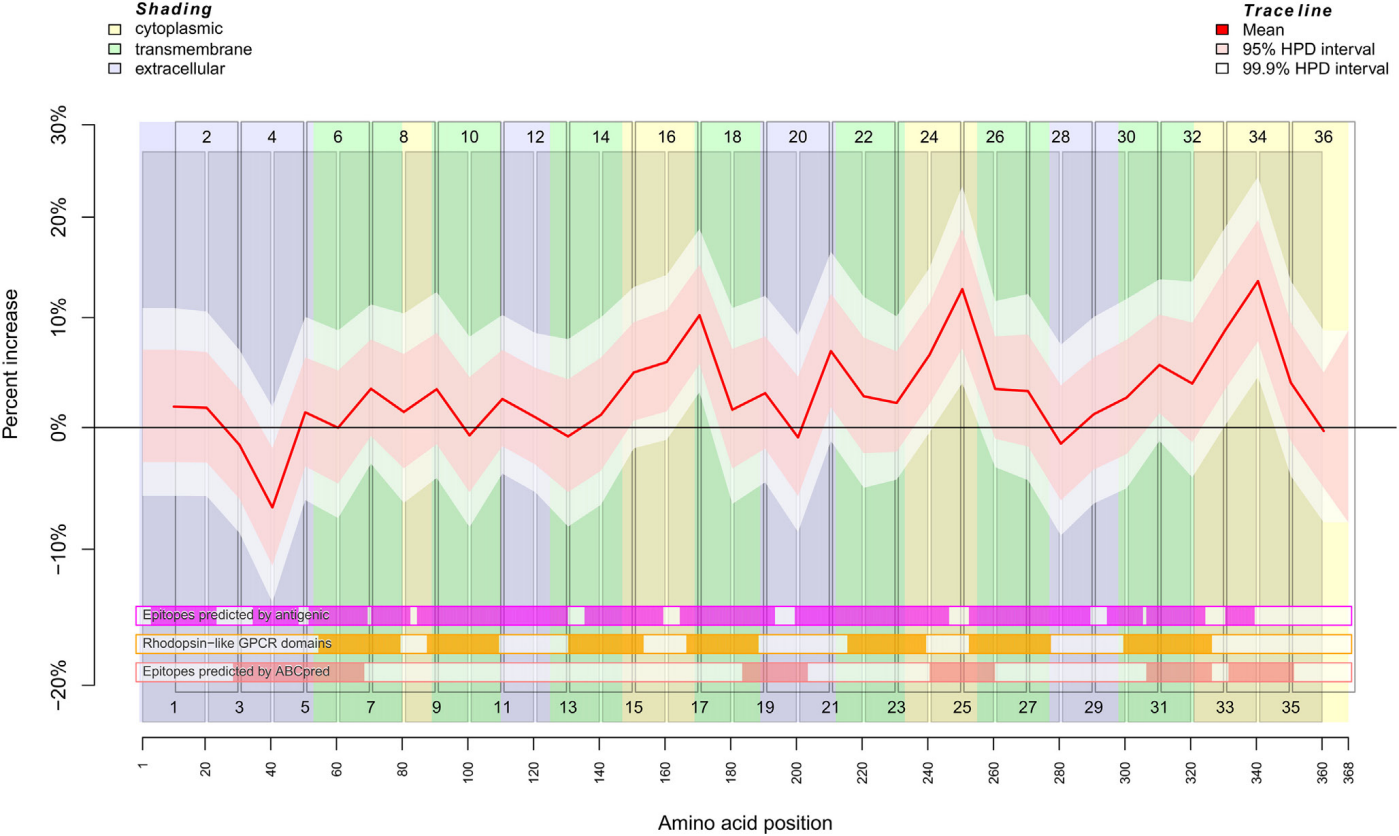
Binding behavior of SSc patient and healthy control sera to individual peptides. Plot of the mean expectation value (red line) and 95 and 99.9% credibility bands (pink and white shading) of the SSc patient-specific ab-binding signal (percent increase). The *x* axis represents amino acid residues 1–368. The peptide localizations are indicated by staggered rectangles that include the peptide numbers. The percent increase indicates the increase or decrease of the binding signal in SSc patients in contrast to an averaged signal. If the 95% (99.9%) credibility interval of the percent increase does not include the zero value (black line), the corresponding peptide is regarded as an epitope that is significantly associated with SSc. By use of the *ar1* model, the percent increase, i.e., the binding signal estimator, for a peptide is influenced by the neighboring peptides. Other fixed and random effects of the statistical model are shown in Figures S2 and S3 in Supplementary Material. At the bottom, three heat maps indicate the position of putative epitopes predicted by *antigenic* (magenta hue), ABCpred (pink hue) and the presence of rhodopsine-like domains (orange hue). The background colors indicate the position of intracellular (yellow hue), transmembranous (green hue), or extracellular (blue hue) amino acid residues.

To separate an SSc-specific binding signal from background noise, we used the Bayesian framework implemented by the *INLA* (Integrated Nested Laplace Approximation) package. Using this R package, we developed a model that incorporates the neighborhood structure, i.e., overlapping of peptides, employing autoregressive models. Non-specific binding of sera and secondary detection antibody to the peptides was modeled by a simple autoregressive model (Figure S2 in Supplementary Material). SSc-specific binding was modeled by combining the autoregressive model with a *weighting* factor (Figure 1). The unspecific binding of serum samples and the inter-plate variability were included as additional random effects. Isoelectric point and hydrophobicity of each peptide were included as fixed effects. These additional random and fixed effects primarily served to remove noise from the SSc-specific binding signal (Figure S3 in Supplementary Material).

**Figure 2:**
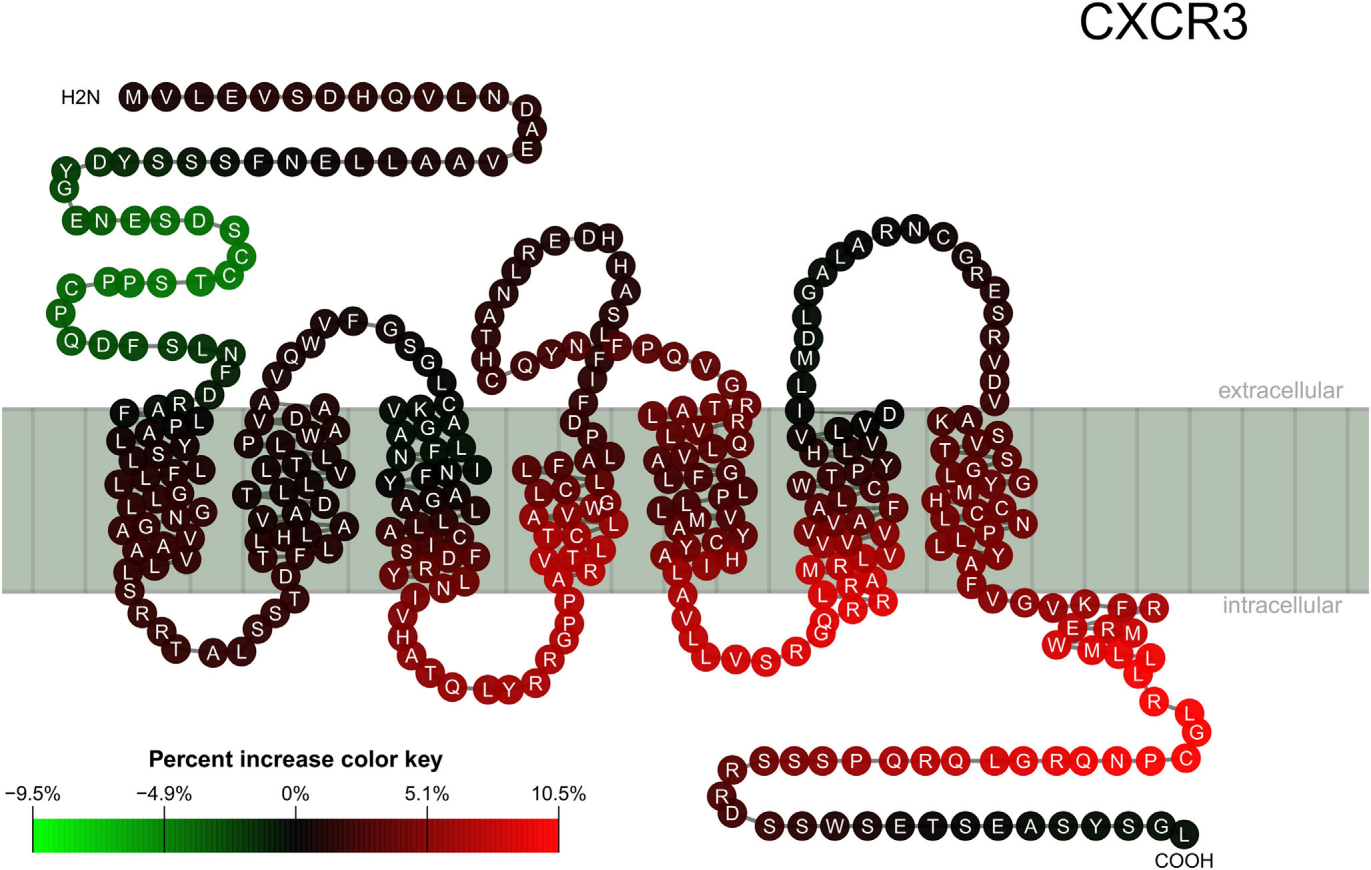
Location of epitopes on the CXCR3 structure. Mapping of binding signal (compare Figure 1) of SSc patient sera to a serpentine model of CXCR3. Each pearl represents a single amino acid residue, the letter inside each pearl the amino acid in 1-letter code. The color of each pearl indicates the percent increase value of the SSc specific binding signal associated with the respective amino acid residue (color key).

The result described in Figure 1 was mapped for visualization to a serpentine model of CXCR3 (Figure 2). For comparison, the logit values of ELISA signals were analyzed peptide-wise with independent classical linear models (Figure S4 in Supplementary Material).

The regression analysis revealed a significant difference between healthy control and SSc patient binding signals for peptide 4 (*p*_Bayes_ = 0.00879), peptide 15 (*p*_Bayes_ = 0.0225), peptide 16 (*p*_Bayes_ = 0.00684), peptide 17 (*p*_Bayes_ = 0.000977), peptide 21 (*p*_Bayes_ = 0.00488), peptide 24 (*p*_Bayes_ = 0.00293), peptide 25 (*p*_Bayes_ = 0.000977), peptide 31 (*p*_Bayes_ = 0.00879), peptide 33 (*p*_Bayes_ = 0.000977), and peptide 34 (*p*_Bayes_ = 0.000977). When using a stricter threshold of *p* < 0.00138 = 0.05/36 to take multiple testing into account, peptides 17, 25, 33, and 34 remain as possible epitopes. In all of these peptides, the binding signal of SSc patients is higher than in healthy controls, except of peptides 4 and 15, which are located at the N-terminal extracellular rod domain. With the exception of peptide 21, all other peptides we identified are located intracellularly.

To compare the reactivity of SSc-abs on peptides with epitopes predicted by the primary structure of CXCR3, we used the EMBOSS *antigenic* software (Figure 1; Figures S2 and S4 in Supplementary Material). This software implements a method described by Kolaskar and Tongaonkar (28). However, there appears to be no true correspondence between predicted and the reactivity of SSc-abs.

In contrast to *antigenic*, the neural network-based *ABCpred* software detected epitopes on amino acid residues Y_29_–L_68_, L_184_–C_203_, V_241_–V_260_, G_307_–R_326_, and L_332_–S_351_ (Figure 1; Figures S2 and S4 in Supplementary Material). From these epitopes, all except L_184_–C_203_ corresponded to peptides we experimentally identified as possible epitopes that differ in the binding between SSc patients and healthy controls. Vice versa, peptides 17 and 21 which were identified by experiment do not correspond to any epitope detected by *ABCpred*. It may be noted that peptide 20 corresponding to *ABCpred* epitope L_184_–C_203_ showed a peak in reactivity in the background signal (Figure S2 in Supplementary Material).

We further checked whether the reactivity of SSc-abs corresponds to conserved Rhodopsin-like GPCR domains (Figure 1; Figures S2 and S4 in Supplementary Material). These domains are mostly located in transmembrane regions of the protein. However, no difference in reactivity between SSc sera and healthy control sera was found in peptides that correspond to Rhodopsin-like GPCR domains.

### A CXCR3 Peptide-Based ELISA Allows Discrimination of SSc and Healthy Control Sera

To verify the epitopes, we detected by the peptide array, we chose peptides 17, 24, 25, 33, and 34 that were positively correlated with SSc sera and combined them into one for coating of ELISA plates. This allowed us to compare 48 samples in duplicates a single microtiter plate. For inter-plate comparison, we used a set of samples as standard samples.

**Figure 3:**
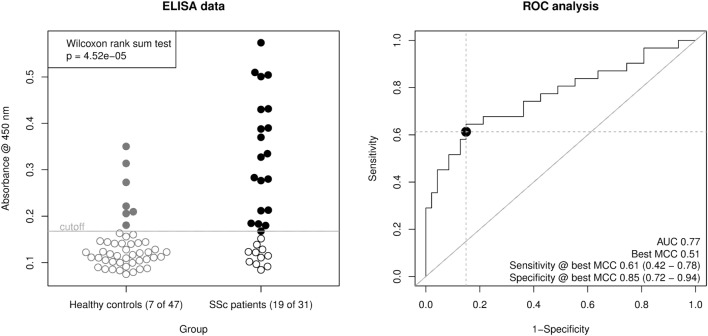
Derivation of a mixed-peptide ELISA for the discrimination of SSc patients and healthy controls. Peptides 17, 24, 25, 33, and 34 were mixed for coating of ELISA plates. In total, 32 SSc patient sera and 47 healthy control sera were measured. Left panel, scatter plot of raw ELISA readout values (OD @ 450 nm) including a cut-off value optimized by maximization of Matthew’s correlation coefficient. SSc patient showed a significantly higher ELISA signal than healthy control sera (Wilcoxon rank sum testing *p* = 4.52 × 10^−5^). Right panel, receiver operator characteristic (ROC) analysis of ELISA values. AUC (area under curve, 0.77) and best MCC (Matthew’s correlation coefficient, 0.51) as well as Sensitivity (0.61) and Specificity (0.85) at the optimal cut-off are shown as performance indicators. The dot indicates the optimal cut-off position in the ROC curve.

Using this ELISA design, we compared raw OD450 values from the 32 SSc patient sera with 16 of the healthy control sera and 31 additional healthy control sera from a new cohort (Figure 3, left panel). The OD450 values of SSc and healthy control sera differed significantly (Wilcoxon rank sum test *p* = 4.52 × 10^−5^). By ROC analysis (Figure 3, right panel), this corresponds to an area under curve (AUC) of 0.77, indicating a good classification ability. A cut-off for the OD450 value could be determined by optimizing Matthew’s correlation coefficient to a value of 0.51. Using this cut-off, the sensitivity of this assay was 0.61 (95% CI: 0.42–0.78), with a specificity of 0.85 (95% CI: 0.72–0.94). This corresponds to a LR+ of 3.98 (95% CI: 2.06–9.28) and a LR− of 0.46 (95% CI: 0.27–0.68).

## Discussion

In this study, we were able to demonstrate that SSc patients’ sera preferentially bind to intracellular epitopes on CXCR3. We furthermore could demonstrate that reactivity to extracellular epitopes is reduced or lost in SSc patients, compared to controls. This especially applies to in the N-terminal rod domain of CXCR3, where a previous study could show that reactivity to this domain is associated with a slower disease progression in SSc (7).

Aabs against GPCRs like CXCR3 have been shown to influence the signaling function of these receptors (5, 9). It is very likely that the epitopes on CXCR3 that are bound by aabs determine different functional and pathophysiological effects. In case of antibodies that bind to receptors the selection of target epitopes decides whether they exhibit activating, inhibiting, internalization-inducing or even neutral (i.e., no measurable) effects (9, 18–20). Peptide arrays provide a straightforward approach to locate linear epitopes recognized by aabs, using ELISA or dot bot methods (20, 29, 30). However, this approach has the drawback that peptides are more flexible in their tertiary structure and tend to bind abs with lower specificity than fully folded proteins. Therefore, a careful statistical evaluation is necessary to detect a specific binding signal (20). This study used an array of 20mer peptides with 10mer overlaps covering the full UNIPROT isoform 1 sequence of CXCR3 to identify linear epitopes bound by aabs in sera from SSc patients and healthy blood donors. The peptide array was employed to conduct a series of ELISA experiments. A highly standardized protocol was used, including anti-human IgG1 as positive process control and a balanced design that always combined sera of an SSc patient and a healthy donor on the same ELISA plate.

To isolate a specific autoantibody-binding signal, we used the Bayesian framework of the R package INLA to design a mixed effects model that separates an autoantibody-related binding signal from inter-experimental variation, variation due to physicochemical properties of individual peptides (hydrophobicity, isoelectric point) and non-specific patient or control serum properties (23, 24). A remarkable advantage of this framework is the possibility to incorporate the neighborhood structure into the model, i.e., the intuitive expectation that two 20mer peptides that have a 10mer overlap should yield a similar signal. Further, the framework provided by the package INLA enabled us to formulate a mathematically more sound model compared to the heuristic approach we used for previous peptide screening studies (20).

Using this mixed effects model, we observed an increased binding of ab from SSc patients to peptides representing intracellular domains of CXCR3, especially the C-terminal rod domain. In contrast, ab binding to the extracellular domains including the N-terminal rod domain appeared to be missing in SSc.

Interestingly, the epitopes we identified did not overlap with epitopes predicted by the *antigenic* software of the EMBOSS bioinformatics package. Only two of the epitopes predicted by *antigenic*, one within the N-terminal rod domain (corresponding to peptide 4) and one within the C-terminal intracellular rod domain (corresponding to peptide 34) appear to overlap. In contrast to *antigenic*, epitope prediction by *ABCpred* showed that 4 of 5 predicted epitope regions correspond to peptides with a binding significantly different between SSc patients and healthy controls. The one epitope predicted by *ABCpred* that showed no differential binding correspond to a peak in the background signal. Although the study is designed to detect epitopes with a reactivity that is different between SSc patients and healthy controls, this indicates that all epitopes predicted by *ABCpred* match our experimental data. The rhodopsin-like GPCR motifs appear to be spared, which might be explained by their higher degree of conservation (31) compared to the other parts of the CXCR3 sequence.

An increased reactivity of abs against an N-terminal fragment of CXCR3 correlated with a more benign progression of lung fibrosis in SSc in an earlier study (7). Thus, we expected a decreased reactivity against linear epitopes within the N-terminal rod domain in our SSc study population, which could indeed be demonstrated with our peptide mapping approach.

To further validate the results from the peptide array, we designed a novel ELISA using a combination of peptides that were found to be associated with SSc. Differently from the peptide mapping approach, where only 2 samples could be processed per microtiter plate, this ELISA allowed to determine 48 samples in duplicates per plate. This allowed a better comparability between samples, as the plate-by-plate variation in ELISA methods is empirically relatively high. The readout of this ELISA allowed discriminating between SSc patient and healthy control sera with a considerably good performance, as expressed by a Matthew’s correlation coefficient of 0.51 and an AUC of 0.77. Most of the healthy control sera used in this mixed peptide ELISA came from an independent cohort that has not been used for identification of epitopes.

We evaluated whether the results from the mixed-peptide ELISA differ between limited and diffuse variants of SSc, which was not the case. However, this novel ELISA concept might be promising for the development of diagnostic tools that allow a better prognosis on deterioration of lung function, pulmonary hypertension, or renal insufficiency and would therefore help to choose the optimal treatment for patients with SSc.

In contrast to extracellular epitopes, the biological relevance of intracellular epitopes—or antigens—is difficult to demonstrate, because they are in general not directly accessible by aabs. ANA, that are typical for collagenoses are directed against intracellular antigens. Although generally regarded as functionally irrelevant, some ANA like anti-Ro may even cross the placenta and cause neonatal lupus (32). It has to be noted here that anti-Ro aabs have been demonstrated to interact with an extracellular epitope of 5-hydroxytryptaminergic (5-HT4) receptor 4 (33), a GPCR like CXCR3. Besides intracellular antigens, intracellular domains of transmembrane proteins have been shown to be targets of aabs, like BP180 (34) and aquaporin-4 (35).

As an exception of the rule that intracellular antigens and epitopes are not accessible by abs, a certain type of aabs has been shown to be able to penetrate the cellular membrane and to bind subsequently to intracellular epitopes and trigger pathogenic mechanisms (36, 37). This observation led to the construction of TransMabs, ab that are designed to penetrate cell membranes using a short (17 aa) membrane translocation sequence (38). In case of anti-DNA mAbs, specific properties of the sequence of the heavy chain complementary-determining regions 2 and 3 appeared to be the prerequisite for their ability to penetrate the cell membrane (37). It may be possible, though challenging to demonstrate, that anti-GPCR aabs may have the ability to penetrate cell membranes and initiate important pathogenic mechanisms by binding to intracellular epitopes.

An interesting finding is the loss of autoreactivity to the N-terminal rod domain of CXCR3 in patients with SSc. Clinically, a lower titer of autoantibodies against the N-terminus of CXCR3 has been associated with a better prognosis of SSc, especially concerning deterioration of lung function (7). Furthermore, aabs against CXCR3 are not only found in SSc, but also in healthy individuals (9). Aabs against GPCRs have been demonstrated to be functionally active (5–9), and it might possible that this is of physiologic importance. Therefore, it appears to be rational to substitute the lacking aabs against CXCR3 and other GPCRs with intravenous immunoglobulins (IVIGs), although only limited evidence exists for a beneficial effect of IVIGs in SSc (39).

In conclusion, we were able to demonstrate that aabs against CXCR3 in SSc patient sera show a different binding pattern like healthy control sera, with increased binding to intracellular epitopes and loss of binding to the extracellular N-terminal rod domain. The results are supported by *in silico* prediction of linear epitopes on CXCR3. Based upon our results, we could devise a novel ELISA concept that may be helpful for monitoring of SSc patients.

## Ethics Statement

All studies with human materials followed the ethical principles established by the Declaration of Helsinki and were approved by the local ethics committee (AZ16-199). All human participants gave their written informed consent.

## Author Contributions

AR designed research, recruited healthy control biomaterials, performed experiments, analyzed data, discussed results, and wrote the manuscript; A-KR performed experiments, analyzed data, discussed results, and wrote the manuscript; FW characterized SSc patients, has analyzed anti-CXCR3 ab in SSc patients, discussed results, and wrote the manuscript; AM recruited patient sera, discussed results, and wrote the manuscript; HH characterized SSc patients and analyzed anti-CXCR3 ab in SSc patients; GM recruited patient sera and kept a biobank; CH discussed results and wrote the manuscript; RL designed research, discussed results, and wrote the manuscript; GR designed research, recruited patient and healthy control biomaterials, discussed results, and wrote the manuscript.

## Conflict of Interest Statement

The authors declare that the research was conducted in the absence of any commercial or financial relationships that could be construed as a potential conflict of interest.
