# Supplementary material for: Autoantibodies in Serum of Systemic Scleroderma Patients: Peptide-Based Epitope Mapping Indicates Increased Binding to Cytoplasmic Domains of CXCR3
Source: Front Immunol. 2018 Mar 22;9:428. doi: 10.3389/fimmu.2018.00428 (PMC5874968; doi:10.3389/fimmu.2018.00428)
Supplement: Supplementary file 1 [file data_sheet_1.docx]

## Supplementary Methods

### Specification of the ELISA data set

The VICTOR 3^TM^ system produces an Excel format output file for each plate it reads, including a sheet with a list including the well number (**A01-H12**) and the raw absorbance at 450 nm in columns. The layout of ELISA plates, i.e. specifications for each well in the plate, was stored in a separate file. Each output file was listed in a reference table file. The whole data set was read using the R package *gdata*, and combined into a flat R data frame (**rawELISAdata**) with the columns **SerumID**, **PlateID**, **Peptide** (peptide number), **Hydrophobicity** of peptide**, pI** (isoelectric point of peptide), **Group** (a numerical value, 1 for SSc, 0 for healthy controls)**.** The logit (**logitABS**) of the absorption value is calculated plate-wise, as described in the methods section. Each row of the flat table contained one measurement of one well.

### R code for INLA model setup

The model was set up using R INLA with default priors for all covariates and hyper-parameters. The R code for the model setup was

Model = inla ( logitABS ~

1 + f(PlateID, model="iid") + f(SerumID, model="iid") +

f(as.numeric(PeptideCopy), model="ar1") +

f(as.numeric(Peptid), Group, model="ar1") +

pI + Hydrophobicity,

data=rawELISAdata, family="gaussian")

Here, **PeptideCopy** is needed as a dummy for **Peptide** because of technical requirements of the R INLA package. The inclusion of the covariates **PlateID**, **SerumID**, **pI** and **Hydrophobicity** into the model are for normalization purposes, while the identification of SSc specific signals are done with the autoregressive models for **Peptide** and **PeptideCopy**.

## Supplementary Information

### Additional remarks on the development of a statistical model for peptide-based epitope mapping

The approach we used for our data analysis is based upon Bayesian models using the INLA approach (23,24). Although Bayesian models are very powerful, they are quite difficult to establish especially in case of hierarchical models. The R package INLA provides a toolbox for model design that overcomes many of these drawbacks.

#### Problem setting

Mapping of linear epitopes of autoantibodies in the primary structure of proteins poses a challenge on data evaluation. Using biotinylated peptides on streptavidin-coated microtiter plates is a straightforward approach, although a sort of standardization is needed to allow comparison of ELISA readouts from different experiments.

In case of peptides, such standardization is not possible, because the peptides representing epitopes that would deliver useful and standardizable signals are unknown beforehand. A possible solution to this is to use a dummy readout which always leads to a positive signal and may be used to monitor color development and compare readouts.

Screening the epitopes of a large protein might require an equivalently large number of peptides that may require a lot space on single microtiter plates to analyze a single sample. Thus, the number of samples that can be analyzed under the same conditions is restricted.

Another source of variance is the non-specific binding of samples to peptides (inter-sample variance) and the variance caused by non-specific binding due to certain physicochemical properties of peptides. Furthermore, sample distribution and washing procedures can cause subtle effects on the ELISA readouts.

Any statistical analysis must be designed to separate the inter-assay variance and non-specific binding from the specific ELISA signal that correlates with the presence of autoantibodies in sample materials.

#### Data transformation

Like many immunological measurement methods, ELISA signals typically follow a sigmoidal curve when mapped to the logarithm of the concentration of the substance that is measured. This sigmoidal relationship can be transformed using the *logit* transformation in order to get a normal distribution of ELISA signals (normalizing transformation).

Furthermore, using ELISA readouts from positive and negative controls of each plate as upper and lower asymptotes, the inter-plate variance may be reduced considerably.

#### The naïve analysis – one-way ANOVA

When measurements of two groups are to be compared, Student’s *t*-test is often a good and popular choice. A one-way ANOVA with two groups is statistically equivalent to the *t*-test.

The one-way ANOVA is based upon a linear model:

$$Y_{i}={\beta_{0}+ \beta_{1}X}_{i} + \epsilon$$

with $Y_{i}$ being the logit of the ELISA signal, $X_{i}$either 1 (disease group) or 0 (control group), $\beta_{0}$ the background signal or intercept and $\beta_{1}$ the group specific signal. $\epsilon$ is an error term. Because the logit value must be exponentiated for recalculation of the ELISA signal, the above shown addition becomes a multiplication. We therefore denominate $\beta_{1}$ as *log factor*. A peptide-wise analysis of our ELISA readout data set, ignoring any of the above described confounding effects, is shown in **Suppl. Fig 4**.

In the Bayesian approach, the values for $\beta_{0}$ and $\beta_{1}$ are considered as random variables. For the model, we need to assume a prior distribution for $\beta_{0}$ and $\beta_{1}$, to calculate the posterior distribution depending on the data. The popular approach of hypothesis testing and calculation of p values is not directly possible (26). However, one may calculate credibility intervals (quantiles or highest-posterior density intervals) for $\beta_{0}$ and $\beta_{1}$ from the posterior distributions and determine the maximum credibility level (e.g. 95%) at which these intervals do not contain the value zero, giving a sort of surrogate for a p value (25,26).

#### Establishing a random-effects model

The core issue with the analysis of peptide-mapping data is missing data: we cannot straightforwardly combine the readout values from different ELISA experiments because of inter-assay variance and the lack of a standard. A solution for this missing data problem is to include as much information about the experimental setup to the model as is possible. Information about the experimental setup is added to the model by adding assumptions about the coefficients. The difficulty here is that these assumptions should not influence the result of the calculation inappropriately or artificially. This is leading to hierarchical random effects models. A framework that provides a comprehensive set of tools to design these random effects models is the mentioned R package INLA (23,24).

A basic information about the peptides is that they are designed in a way that two 20mer peptides in a sequence have an overlap of 10 amino acids. It is therefore likely, that the ELISA signals of two overlapping peptides are correlating. Such a setting corresponds to a combination of two latent models - autoregressive models of order 1 - of the INLA system, one for the intercept $\beta_{0}$and one for the coefficient $\beta_{1}$ that is related to the disease group (SSc vs. healthy control). In both cases, the coefficients ($\beta_{0}$or$\beta_{1}$) corresponding to a peptide must resemble the coefficients of the neighboring peptides. The degree of resemblance is actually data-driven. In other words, the amount of information we can take from neighboring peptides for calculating the coefficient of a single peptide is determined by the data itself and does not require further assumptions. It has to be mentioned here that the assumptions in Bayesian models are formulated as prior distributions for the coefficients. Hierarchical models add a second layer, using hyper-parameters with their own prior distribution, to describe the prior distributions of the coefficients. We used the default vague priors to avoid artificial skewing of posterior distributions for the coefficients.

It has to be noted that we are still working with the simple linear model formula described above, only with some additional constraints put on the coefficients.

Hierarchical models have the property denoted as *shrinkage*. Depending on the amount of information within the data, the shrinkage effect might even level out the differences between the model coefficients governed by a latent model, e.g. for the different peptides. This property is very useful during model development and data exploration, because covariates that do not bear information important for the response variable can be easily identified. Additionally, the deviance information criterion can be used to identify the best model. The best model has the lowest DIC.

To take other noise factors into account, we included additional latent models that reflect plate-by-plate variation, serum-by-serum variation and variation due to physicochemical properties of the peptides (**Suppl. Fig. 3**).

Based upon these considerations, we compared a whole set of models (**Suppl. Tab. 1**) and chose that one which has the lowest DIC and does not contain covariates that were leveled out by shrinkage.

## Supplementary Tables

### Supplementary Table 1 – Comparison of different models for the peptide mapping using the deviance information criterion (DIC)

| #^1^ | Covariates^2^ | | | | | | | | | | | | | DIC |
| --- | --- | --- | --- | --- | --- | --- | --- | --- | --- | --- | --- | --- | --- | --- |
|  | Disease Group | Peptide | Peptide^3^ with Disease Group | Plate ID | Serum ID | pI | Hydro-phobicity | Serum ID^3^  with Disease Group | Plate ID^3^ with Disease Group | ABCpred | ABCpred^3^ with Disease Group | Peptide^3^ with ABCpred | Peptide^3^ with ABCpred with Disease Group |  |
| 1 |  | *ar1* model | *ar1* model | *iid* model | *iid* model | fixed | fixed |  | *iid* model |  |  | *ar1* model | *ar1* model | -5838.353 |
| 2 |  | *ar1* model | *ar1* model | *iid* model | *iid* model |  |  | *iid* model | *iid* model |  |  |  |  | -5837.589 |
| 3* |  | *ar1* model | *ar1* model | *iid* model | *iid* model | fixed | fixed |  |  |  |  |  |  | -5837.555 |
| 4 |  | *ar1* model | *ar1* model | *iid* model | *iid* model | fixed | fixed |  | *iid* model |  |  |  |  | -5837.135 |
| 5 |  | *ar1* model | *ar1* model | *iid* model | *iid* model | fixed | fixed |  |  | *rw1* model | *rw1* model |  |  | -5836.861 |
| 6 |  |  |  | *iid* model | *iid* model | fixed | fixed |  | *iid* model | *rw1* model | *rw1* model |  |  | -5483.819 |
| 7 | fixed |  |  | *iid* model | *iid* model | fixed | fixed |  | *iid* model | fixed | fixed |  |  | -5210.307 |
| 8 | fixed | *ar1* model | *ar1* model |  |  |  |  |  |  |  |  |  |  | -4648.972 |
| 9 | fixed | *ar1* model | *ar1* model |  |  | fixed | fixed |  |  |  |  |  |  | -4647.974 |
| 10 | fixed | fixed | fixed |  |  | fixed | fixed |  |  |  |  |  |  | -4246.471 |
| 11 | fixed | fixed | fixed |  |  |  |  |  |  |  |  |  |  | -4639.31 |

1. Model number with DIC in ascending order. The model (*****) that was finally chosen is highlighted in red. The general rule is: the lower the DIC, the better the model.
2. *Disease group* is a dichotomous variable; *Peptide*, *Serum ID* and *Plate ID* categorical variables; *pI* and *Hydrophobicity* continuous variables; *ABCpred* is a continuous variable between [0;1], indicating the amount of amino acids in a peptide that are part of an epitope predicted by the ABCpred method
3. Interactions are either encoded as fixed effects (always combined with interacting variables in separate) or, which is mathematically equivalent, as weights in *iid*, *rw1* and *ar1* models, with the 1^st^ variable denoted in the headers as primary variable for the model setup and the 2^nd^ or the product of the 2^nd^ and 3^rd^ variable as weights.

## Supplementary Table 2

| Column Header | Data type | Comment |
| --- | --- | --- |
| Plate.ID | Integer | Identifier for individual ELISA plates, i.e. separate experiments |
| Well | Factor **A01**-**H12** | ELISA well |
| ABS.450nm | Numeric real | Absorption at 450 nm |
| Normalized.ABS | Numeric real | By-plate normalization (see methods) |
| Logit.ABS | Numeric real | Absorption logit value (see methods) |
| Serum.ID | Factor | Unique identifier for each serum sample in the study |
| Group | Factor **Patient** or **NHS** | Each serum belongs to either the SSc patient (**Patient**) or the healthy control (**NHS**) group |
| Peptide | Factor **1**-**36** or *NA* | Peptide number, NA for controls |
| Start^1^ | Integer | Amino acid residue that corresponds to the **first** amino acid of the peptide (see **Tab. 2**) |
| End^1^ | Integer | Amino acid residue that corresponds to the **last** amino acid of the peptide (see **Tab. 2**) |
| Coating | String | A comment on the coating. Identical to Peptide, but with some more information about (i) the solvent used to dissolved the peptide - standard or alternative – and (ii) positive and negative controls are indicated. |
| Hydrophobicity^1^ | Numeric real | Calculated hydrophobicity score (see **Tab. 2**) |
| pI^1^ | Numeric real | Calculated isoelectric point (see **Tab. 2**) |
| Charge^1^ | Numeric real | Calculated charge of the peptide at pH 7.2 |
| Extracellular^1^ | Numeric real, 0 to 1 | Amount of amino acids in the peptide that correspond to extracellular locations of the CXCR3 protein |
| Transmembrane^1^ | Numeric real, 0 to 1 | Amount of amino acids in the peptide that correspond to transmembrane locations of the CXCR3 protein |
| Cytoplasmic^1^ | Numeric real, 0 to 1 | Amount of amino acids in the peptide that correspond to cytoplasmic llocations of the CXCR3 protein |
| Rhodopsin^1^ | Numeric real, 0 to 1 | Amount of amino acids in the peptide that correspond to extracellular locations of the CXCR3 protein |
| Antigenic.predicted.epitope^1^ | Numeric real, 0 to 1 | Amount of amino acids in the peptide that correspond to epitopes predicted by *antigenic* |
| ABCpred.predicted.epitope^1^ | Numeric real, 0 to 1 | Amount of amino acids in the peptide that correspond to epitopes predicted by *ABCpred* |
| Experiment.Day | Factor | Day of experiment |

1. These entries are dependent on the peptide. For controls, the values are set to *NA* (not available).

## Supplementary Figures

### Supplementary Figure S1 – Heat-map of raw peptide mapping ELISA values

Heat-map of raw ELISA readouts from the peptide mapping experiments. Each row corresponds to a single serum sample, the SSc patient sera were ordered at the top of the map. Each column corresponds to a single peptide. The intensity of the yellow color indicates the strength of the signal. Repeated measurements were averaged using the median.

### Supplementary Figure S2 – Unspecific binding of sera to individual peptides

Plot of the mean expectation value (red line) and 95% and 99.9% credibility bands (pink and white shading) of the unspecific binding signal (**percent increase**). The x axis, background shading and heat maps on the bottom are identical to **Fig. 1**. The INLA calculation separates the specific binding signal of SSc patients’ sera and the binding signal of all sera. In healthy controls, the ELISA signal is proportional to the unspecific signal shown in this figure. In SSc patients, the ELISA signal is proportional to the unspecific signal multiplied with the SSc specific signal shown in **Fig. 1**. The values shown in this figure describe the binding behavior of peptides to all sera in general, irrespective of whether the serum is from patients or healthy controls.

### Supplementary Figure S3 – Posterior distributions of included model covariates

To separate variation caused by the measurement process, non-specific peptide properties and sample from the specific signal of aab binding, additional covariates are included as random and fixed effects. **A**, probability density function (p.d.f.) plot of the posterior distribution of the model’s **Intercept**. The x axis corresponds to the coefficient value (increase (in percent)). **B**, p.d.f. plot of the posterior distribution of the increase (in percent)of isoelectric point (**pI**). **C**, p.d.f. plot of the posterior distribution of the increase (in percent) of hydrophobicity score (**Hydrophobicity**). In **A**, **B** and **C**, the Bayesian 95% highest-posterior density (HPD) credibility interval is indicated by the gray shading. The credibility interval in **C** does not contain the zero point, which indicates a significant contribution of the hydrophobicity to a decreased binding of sera to hydrophobic peptides. **E**, violin plots of the posterior distribution of the log factors associated with the **PlateID**, corresponding to the plate-by-plate or inter-assay variation. **E**, violin plots of the posterior distribution of the log factors associated with the **SerumID**, corresponding to the sample-by-sample or non-specific inter-individual variation. The violin bulk of some of the samples are clearly deviating from the zero line, indicating samples with increased non-specific binding signals. **F**, boxplot PIT values (leave-one-out cross-validation output) for individual measurements aggregated by serum samples. The PIT value is the probability that the response value (ELISA readout) predicted by the model (after leaving out the respective value) is larger than the original response value. A PIT value around 0.5 is optimal.

### Supplementary Figure S4 – Simplified analysis of peptide mapping results

Plot of the mean expectation value (red line) and 95% and 99.9% confidence bands (pink and white shading) of the SSc specific binding signal (**percent increase**). The x axis, background shading and heat maps on the bottom are identical to **Fig. 1**. The SSc specific binding signal is calculated using a linear model applied individually to each peptide, ignoring all other sources of experimental variation.

## Supplementary Data Set

A raw data table containing all data from the peptide mapping array that has been used in the presented analyses. Each data row corresponds to a single ELISA well. The columns are specified in **Suppl. Tab. 2.**
