# Supplementary figures and images for: Autoantibodies in Serum of Systemic Scleroderma Patients: Peptide-Based Epitope Mapping Indicates Increased Binding to Cytoplasmic Domains of CXCR3
Source: Front Immunol. 2018 Mar 22;9:428. doi: 10.3389/fimmu.2018.00428 (PMC5874968; doi:10.3389/fimmu.2018.00428)

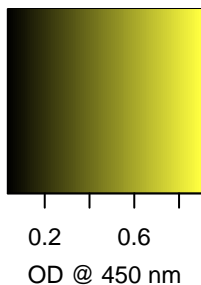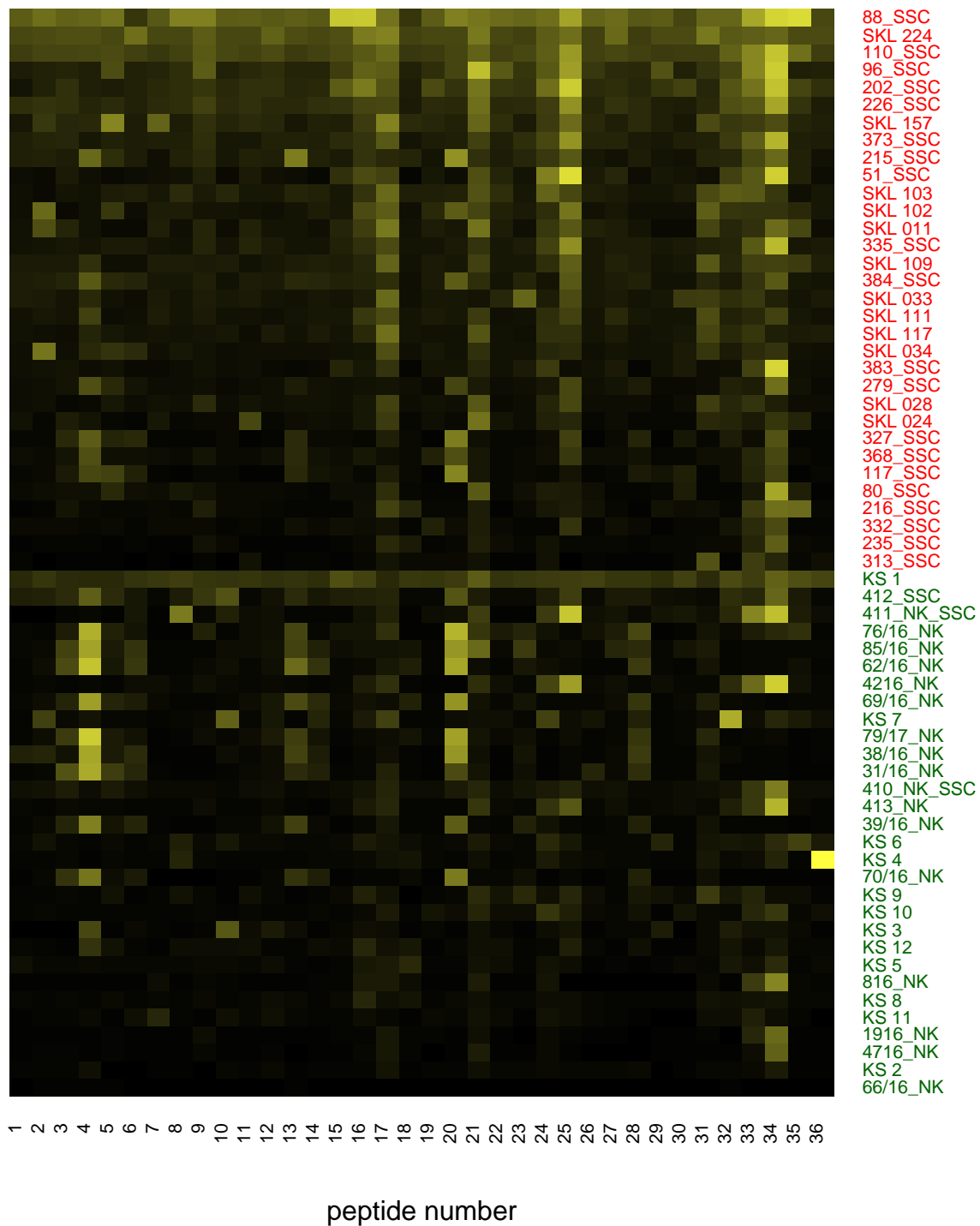

Supplement: Supplementary file 3 [file image_1.PDF]

**Shading**

- cytoplasmic
- transmembrane
- extracellular

**Trace line**

- Mean
- 95% HPD interval
- 99.9% HPD interval

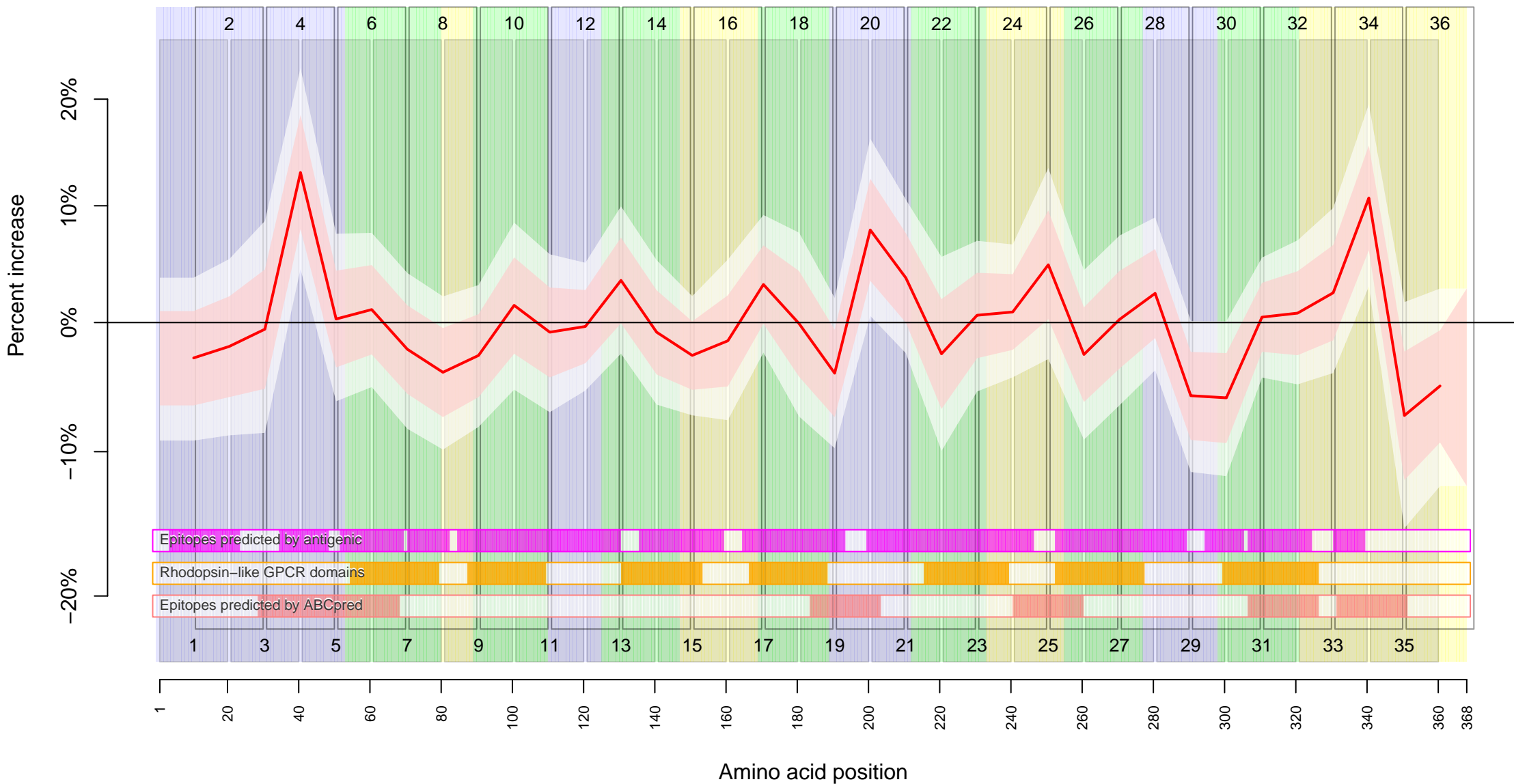

Supplement: Supplementary file 4 [file image_2.PDF]

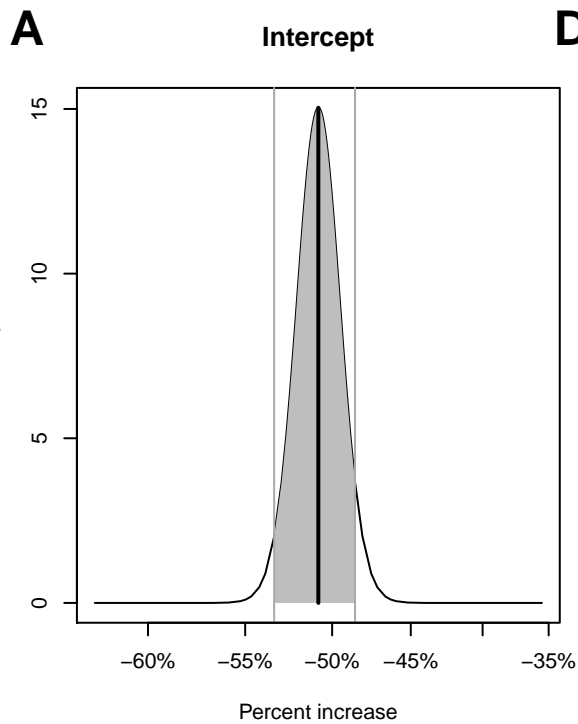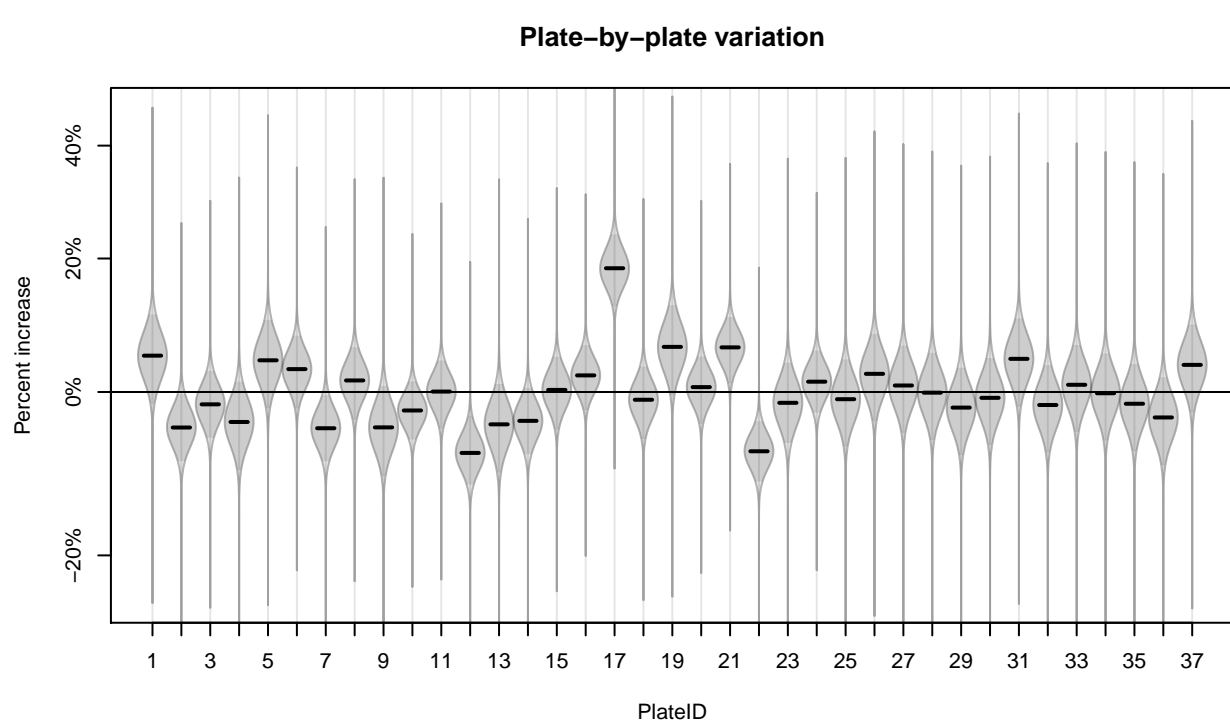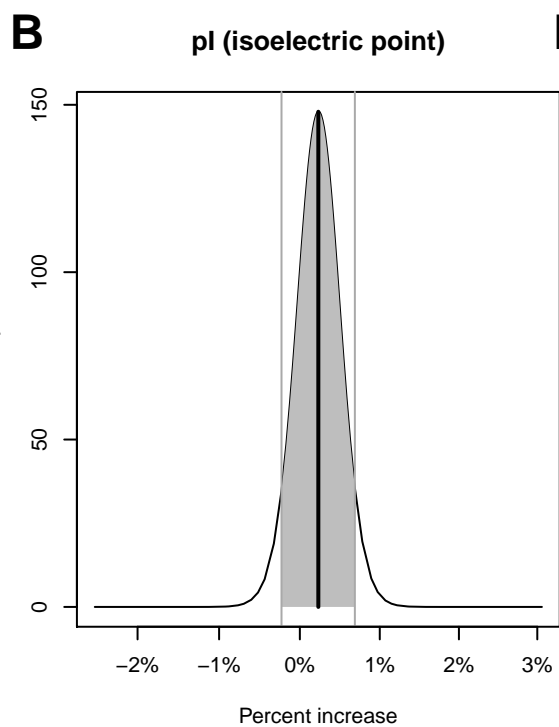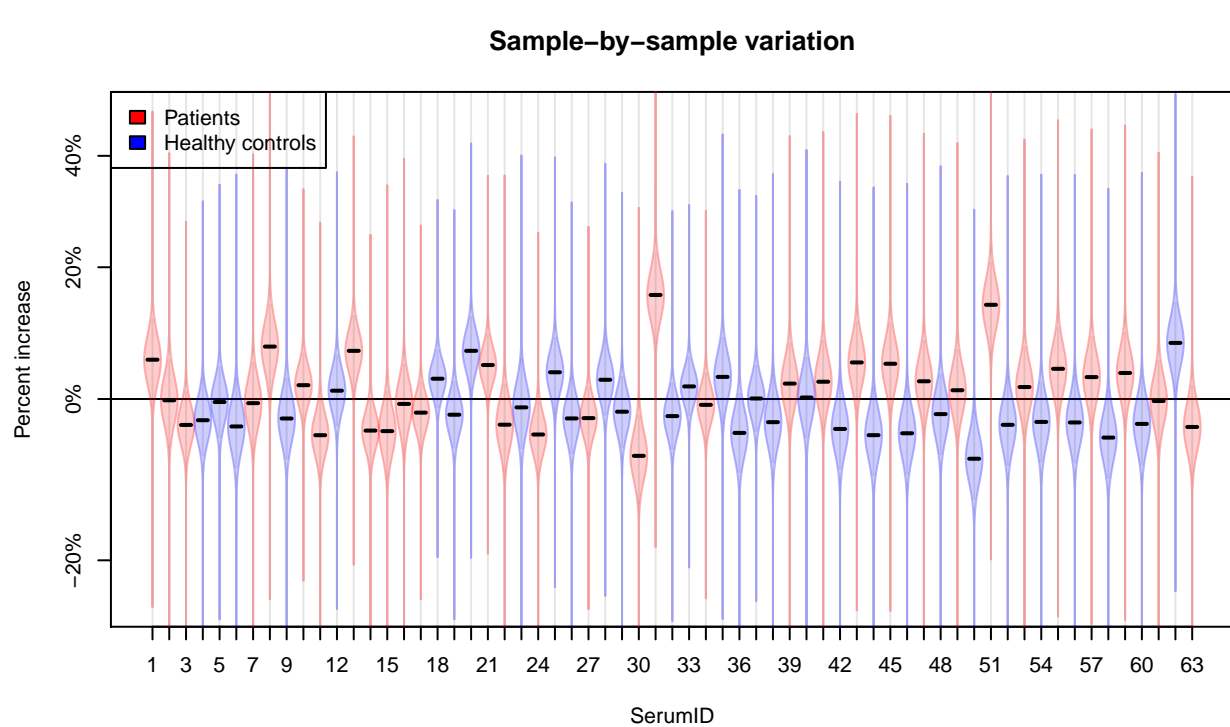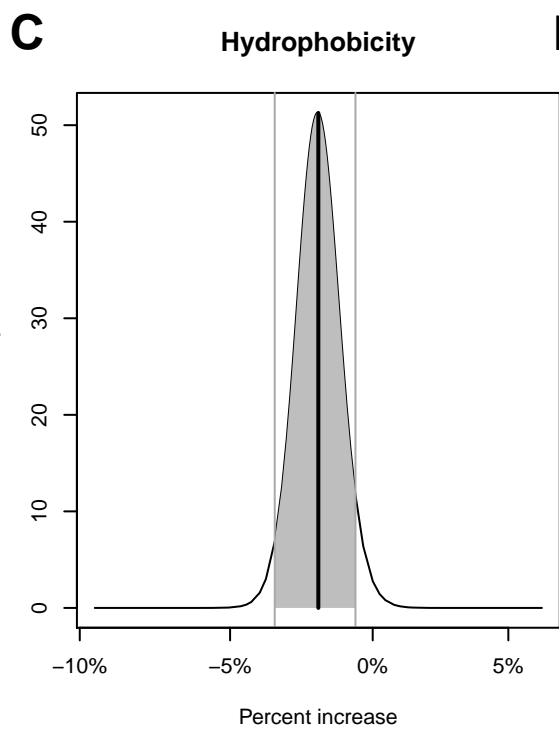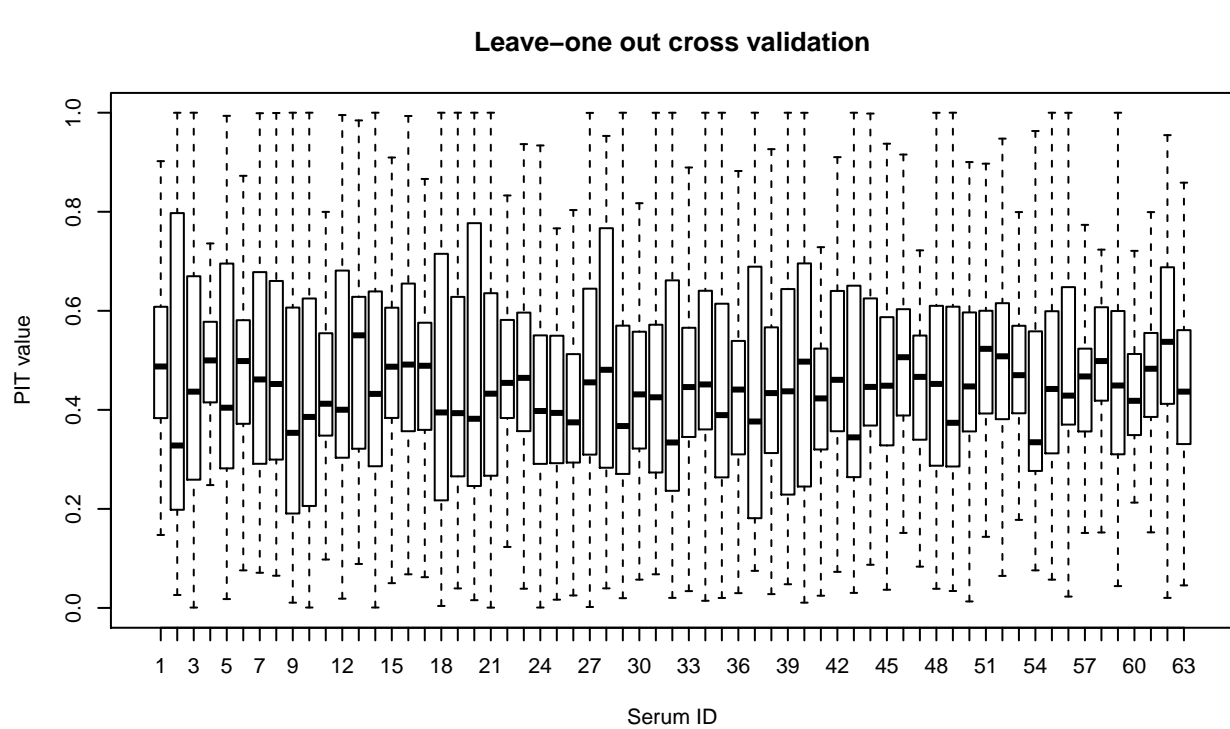

Supplement: Supplementary file 5 [file image_3.PDF]
