# Supplementary material for: Autoantibodies in Serum of Systemic Scleroderma Patients: Peptide-Based Epitope Mapping Indicates Increased Binding to Cytoplasmic Domains of CXCR3
Source: Front Immunol. 2018 Mar 22;9:428. doi: 10.3389/fimmu.2018.00428 (PMC5874968; doi:10.3389/fimmu.2018.00428)
Supplement: Supplementary file 6 [file image_4.PDF]

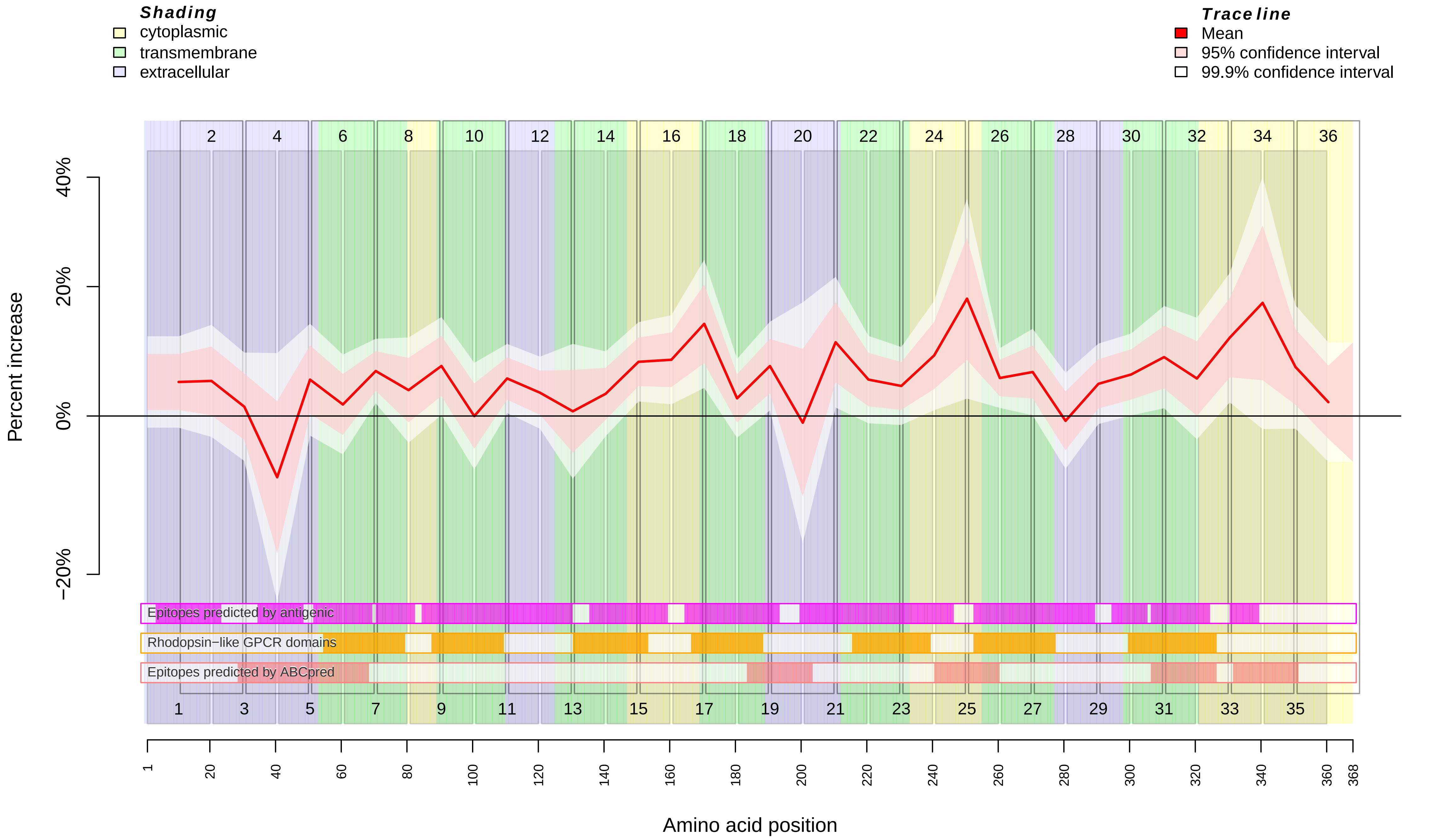
Shading
cytoplasmic
transmembrane
extracellular
Trace line
Mean
95% confidence interval
99.9% confidence interval
Percent increase
40%
20%
0%
−20%
Epitopes predicted by antigenic
Rhodopsin−like GPCR domains
Epitopes predicted by ABCpred
Amino acid position
